# Supplementary material for: Adipose Tissue-Derived Stem Cells Retain Their Adipocyte Differentiation Potential in Three-Dimensional Hydrogels and Bioreactors
Source: Biomolecules. 2020 Jul 17;10(7):1070. doi: 10.3390/biom10071070 (PMC7408056; doi:10.3390/biom10071070)
Supplement: Supplementary file 1 [file biomolecules-10-01070-s001.zip › Supplementary Figures Revised/Figure S1 Matlab Code.pdf]

Figure S1: MATLAB Code to Determine Calcein Positive Stating

```
%% Set up
clc, close all, clear all
%% Data Read
filename='LiveDead_Bioreactor_Con_4x'; %Picture Name
I_cmyk=imread([filename,'.tif']); %Read in image
I_rgb=I_cmyk(:,:,1:3); %All transperancy of images are equal to 255, can make
direct conversion
S=size(I_rgb);
IR_O=zeros(S);
IG=I_rgb(:,:,2); %Pull out GFP channel
IB=I_rgb(:,:,3); %Pull out background channel (blue)
IR=IG-IB; % subtract background
%% Thresholding out background staining
I_eq = imadjust(IR);
bw_grayThresh = im2bw(I_eq, graythresh(I_eq));
% attempt with different thresholding
bw_adaptThresh = imbinarize(I_eq,'adaptive','Sensitivity',0.65);
I_subtracted = I_eq;
I_subtracted(~bw_adaptThresh) = 0;
I_subtracted(~bw_grayThresh) = 0;
IR_Th=I_subtracted;
%% Watershed Hist
gmag = imgradient(IR_Th);
L = watershed(gmag);
Lrgb = label2rgb(L);
%% Morpholigcal operations to determine cell area
se = strel('sphere',3);
Io = imopen(IR,se);
Ie = imerode(IR,se);
Iobr = imreconstruct(Ie,IR);
Ioc = imclose(Io,se);
Iobrd = imdilate(Iobr,se);
Iobrcbr = imreconstruct(imcomplement(Iobrd),imcomplement(Iobr));
Iobrcbr = imcomplement(Iobrcbr);
fgm = imregionalmax(Iobrcbr);
I2 = labeloverlay(I_rgb,fgm);
%% Determing properties of identified regions of interest
LiveInfo=regionprops(fgm,'area');
[x,y]=size(IR);
cellArea(:,1)=[LiveInfo.Area];
sizeArea=size(cellArea);
totalArea=x*y;
range=['A',num2str(sizeArea(1)),':B1'];
cellArea(1,2)=totalArea;
%% Showing image with overlayed area to check results
%gm=fgm-bw;
figure(1)
title('Original')
imshow(I_rgb)
bw_perim=bwperim(fgm);
overlay1=imoverlay(I_rgb, bw_perim, [.3 1 .3]);
figure(2)
title('segemented image')
imshow(overlay1)
%% Export to excel
writematrix(cellArea,filename,'FileType','spreadsheet','Range',range)
```
