# Supplementary figures and images for: Adipose Tissue-Derived Stem Cells Retain Their Adipocyte Differentiation Potential in Three-Dimensional Hydrogels and Bioreactors
Source: Biomolecules. 2020 Jul 17;10(7):1070. doi: 10.3390/biom10071070 (PMC7408056; doi:10.3390/biom10071070)

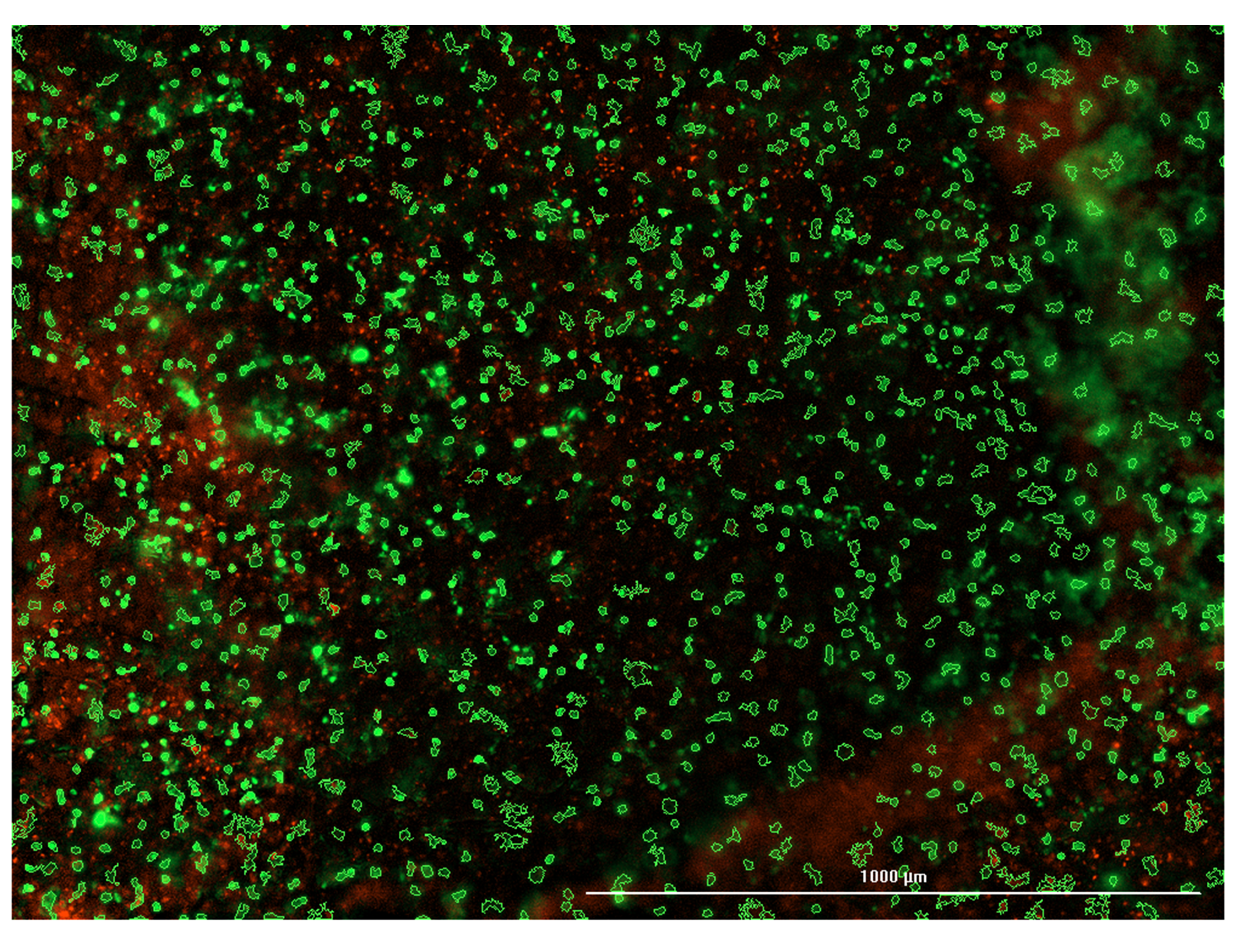

Supplement: Supplementary file 1 [file biomolecules-10-01070-s001.zip › Supplementary Figures Revised/S2.TIF]

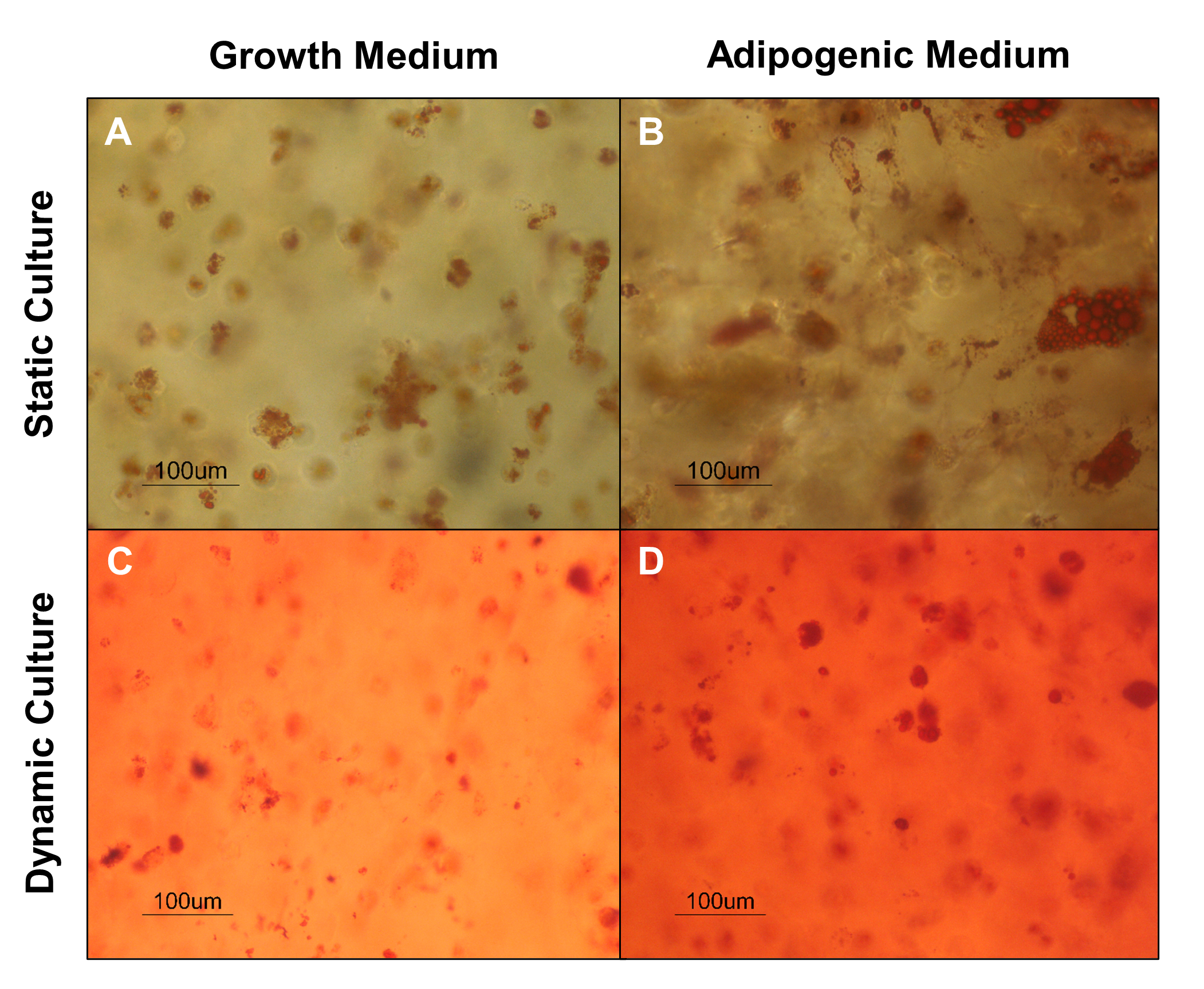

Supplement: Supplementary file 1 [file biomolecules-10-01070-s001.zip › Supplementary Figures Revised/S3.TIF]

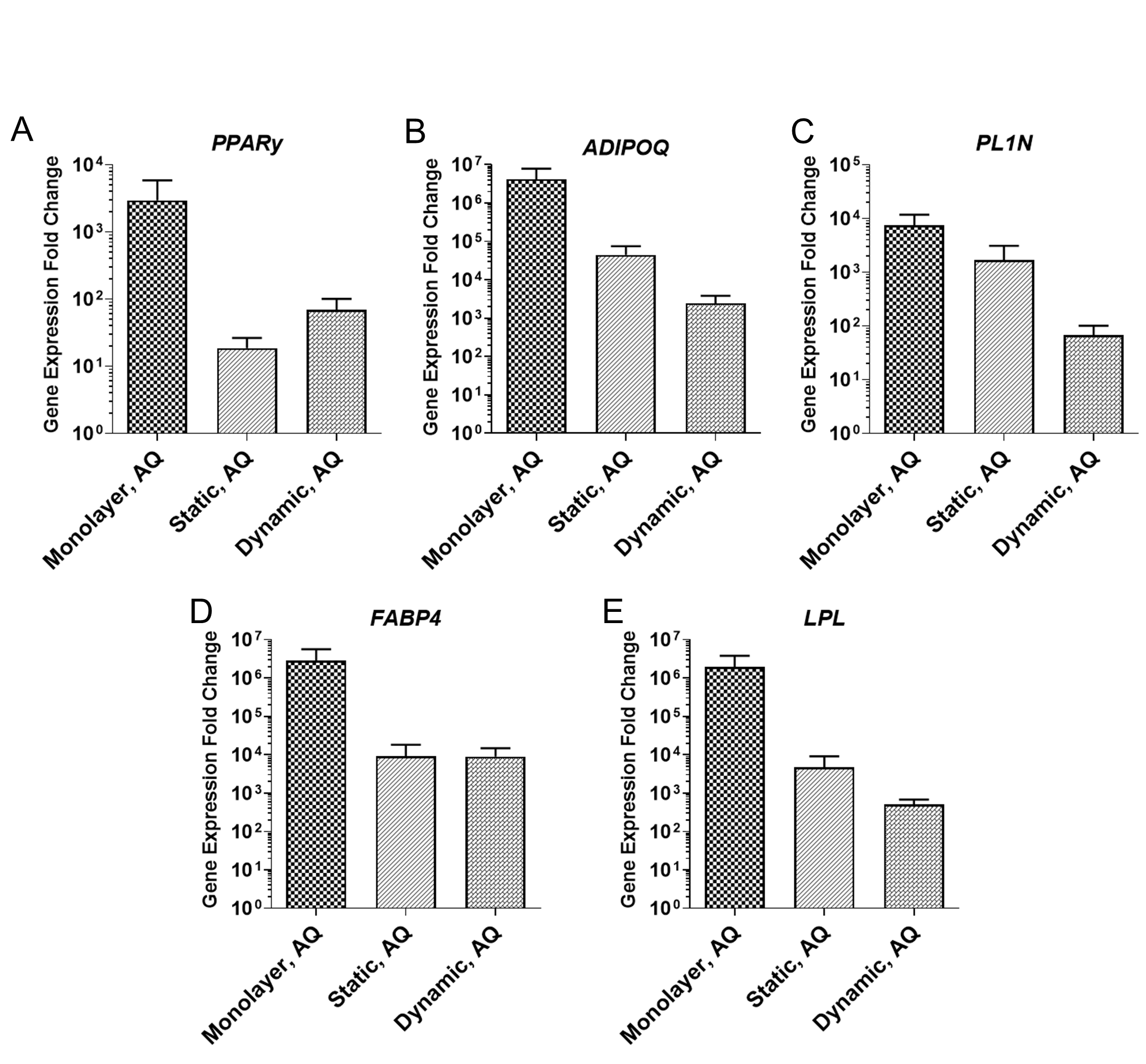

Supplement: Supplementary file 1 [file biomolecules-10-01070-s001.zip › Supplementary Figures Revised/S4.TIF]

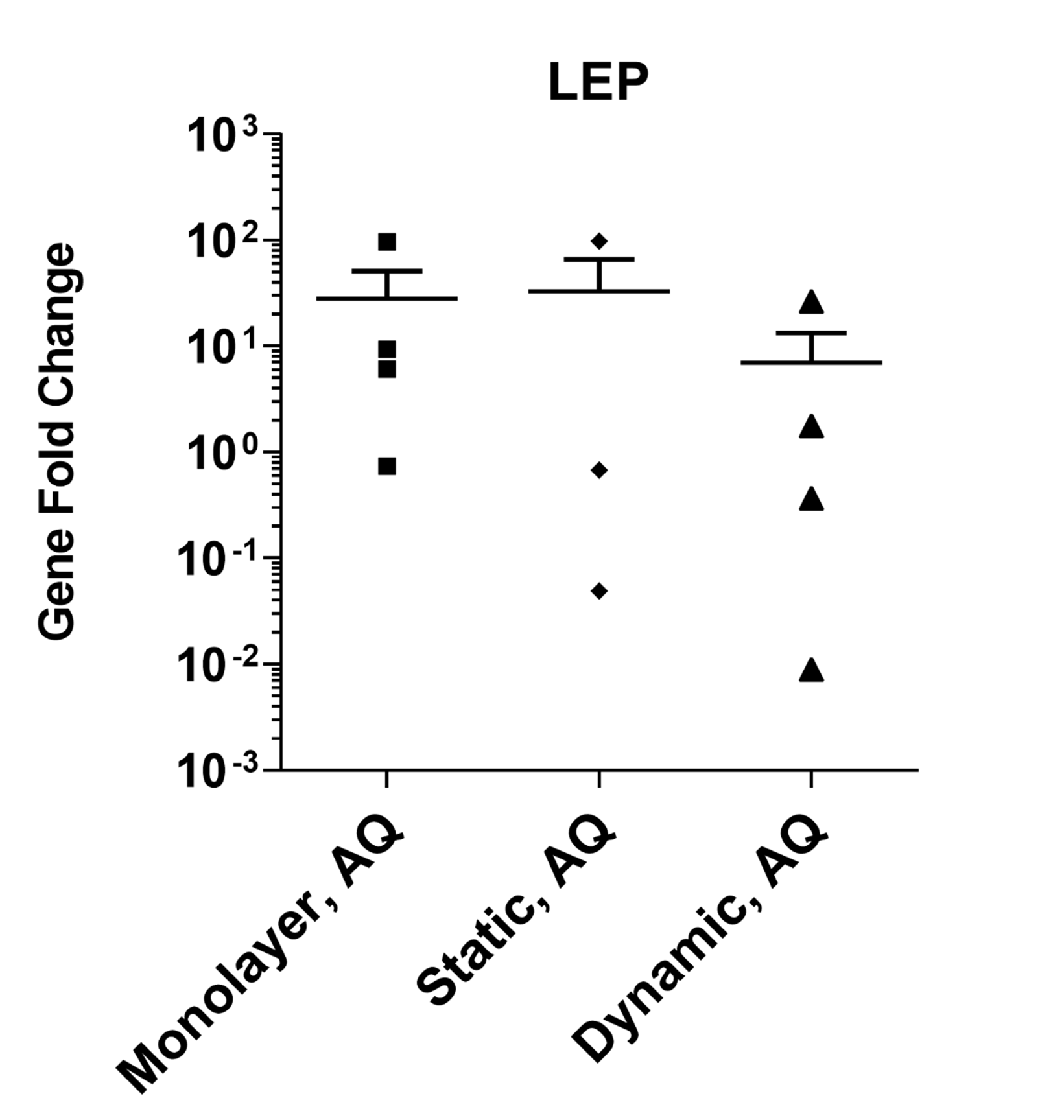

Supplement: Supplementary file 1 [file biomolecules-10-01070-s001.zip › Supplementary Figures Revised/S5.TIF]
